# Treatment of Heyde’s Syndrome by Aortic Valve Replacement

**DOI:** 10.2174/157340311795677699

**Published:** 2011-02

**Authors:** Ramzi Abi-akar, Issam El-rassi, Nicole Karam, Yehya Jassar, Rita Slim, Victor Jebara

**Affiliations:** Saint Joseph University, School of Medicine Beirut, Lebanon

**Keywords:** Aortic valve, angiodysplasia, von willebrand, gastrointestinal hemorrhage, bleeding.

## Abstract

Heyde’s syndrome is the association between calcific aortic stenosis and gastrointestinal bleeding due to angiodysplasia. Alterations in von Willebrand factor due to turbulence across the diseased aortic valve have been incriminated in the pathophysiology of this syndrome. Replacement of the aortic valve has been reported to stop the bleeding, but this is debatable. Along with a review of the relevant medical literature, we hereby report a 68 year old patient with aortic stenosis and severe recurrent gastrointestinal bleeding that completely subsided following aortic valve replacement.

## INTRODUCTION

Heyde’s syndrome is the association between calcific aortic stenosis and gastrointestinal bleeding due to angiodysplasia. Many questions regarding its pathophysiology and treatment remain unanswered. Whether aortic valve replacement may stop gastrointestinal bleeding is still controversial, although it was suggested as early as 1974 as a first line treatment for this syndrome [1,2]. We hereby review the literature, and report a case of severe gastrointestinal bleeding from gastrointestinal tract angiodysplasia associated with aortic valve stenosis, successfully treated by aortic valve replacement.

## CASE REPORT

A 68 year-old male, was admitted to the hospital in march 2008 with a history of melena. His past medical history included several hospitalizations for severe episodes of anemia due to gastrointestinal bleeding that required multiple endoscopic interventions on the colon, with plasma Argon coagulation and blood transfusion. Endoscopic examinations showed multiple sites of angiodysplasia of the duodenum and of the colon. Further evaluation with an endoscopic capsule revealed multiple arteriovenous malformations of the intestine, especially in the jejunum with no evidence of tumors or polyps. He was also known to have a moderate degenerative calcified aortic stenosis. Repeat echocardiography revealed severe aortic stenosis with a mean gradient of 50 mmHg, and an orifice area of 0.8 cm^2^. Moderate aortic insufficiency was also noted. Although he was advised to undergo aortic valve replacement, the patient refused the operation due to the potential intestinal hemorrhage secondary to anticoagulation. The relation between intestinal angiodysplasia and aortic valve disease was not made.

The patient continued to suffer from intestinal bleeding requiring several transfusions. His exercice tolerance decreased dramatically over the months and he was admitted in May 2009 to undergo aortic valve replacement. Blood testing showed a hemoglobin level of 11g/dl (two weeks following transfusion of two packed cells units) and a platelets count of 128000/mm^3^. Values of bleeding time, prothrombin time, Ristocethin cofactor activity, Von Willebrand and factor VIII levels were all in the normal range. Von Willebrand factor was measured *via* gel electrophoresis. Echocardiography confirmed progression of his aortic stenosis with a mean pressure gradient of 66 mmHg, a maximal gradient of 97mmHg and a functional area of 0.7cm2. Coronary angiography revealed severe stenosis of the left anterior descending and the right coronary artery. 

Under standard cardiopulmonary bypass, aortic valve replacement and two coronary artery bypass grafts were performed. In order to avoid long term oral anticoagulation, a 23mm Carpentier-Edwards pericardial bioprosthesis was chosen. The postoperative course was uneventful. There was no intestinal bleeding. 

He was discharged on Beta-blocker, diuretics, and coumadin which was discontinued after three months. During the 9 months follow-up the patient showed no signs of intestinal bleeding and required no transfusions. His Hemoglobin level at this time is 13 g/dl.

## COMMENTS

The association between aortic valve stenosis and intestinal angiogysplasia was first described in 1958 by Heyde and was subsequently referred to as Heyde’s syndrome [3]. However, this association remains controversial, as opposing conclusions have been reached by several retrospective studies and cohort series [4-6].

Finding statistical proofs or causal links between aortic stenosis and intestinal dysplasia has been extremely difficult because they are both common in the elderly; age is a statistically significant confounding factor, as patients who had been diagnosed with both conditions were older than patients with only one or neither (p<0.0001) [7]. Hence, the association between these two conditions might only be a coincidence due to the concommittant senile degeneration of both the aortic valve and intestinal mucosa [2]. However, syndrome or coincidence, this combination constitutes a real threat to the elderly and its stormy complications cannot be overlooked.

In 1971, Boss and Rosenbaum described distended vessels in the intestinal mucosea in the post mortem examination of a patient with aortic stenosis [8]. They theorized that this may have been the result of chronic low grade hypoxia, causing sympathetic vasodilation and smooth muscle relaxation progressing to true ectasia of vessel walls. Others have suggested that intestinal mucosal ischemia, especially the colonic mucosae, may be due to the altered pulse waveform in aortic stenosis, or may be caused by cholesterol crystal embolisation from the aortic valve [9].

Abnormalities of von Willebran factor have also been incriminated as a major cause of bleeding in this syndrome [5, 10]. The bleeding disorder may be explained by an acquired type IIA von Willebrand ‘s syndrome, which is a deficiency of high molecular weight multimers of von Willebrand factor (VWF) [11-15]. It is probably a result of an accelerated interaction with platelets due to turbulent blood flow through the stenotic valve: This increases the breackdown of high molecular weight multimers of VWF and could be corrected by surgery. It had been demonstrated recently that the loss of large VWF multimers affects both platelet adhesion, and ADP-inducible platelet aggregation [16]. Hence, VWF is more important than other clotting factors for hemostasis in abnormal vessels such as angiodysplasia and telangiectasia [6, 17]. In a case controle study, Veyradier and colleagues showed variable selective loss of the largest multimeric forms of von Willebrand factor in eight out of nine patients with bleeding digestive angiodysplasias or telangiectasias. Seven of these patients had aortic stenosis. In contrast, patients with nonbleeding angiodysplasias had normal VWF profiles [18]. This may explain why von Willebrand abnormalities and bleeding seem to disappear following aortic valve replacement and re-replacement [13].

Acquired von Willebrand factor-IIA abnormalities may be detected preoperatively. Von Willebrand factor ristocetin cofactor activity and von Willebrand factor antigen level are the least sensitive tests. Measuring the levels of high molecular weight multimers of von Willebrand factor, and dynamic platelet function testing may reveal the coagulopathy [9]. However, the gold standard exam is gel electrophoresis of von Willebrand factor, showing the absence of large polymers [4].

With persistent anemia requiring multiple blood transfusions, aortic valve replacement should be considered, even in asymptomatic patient with a hemodynamically significant aortic stenosis [2].

However, some reports don’t favor this strategy as the best treatment option. In a letter to the American Journal of Gastroenterology, Bhutani and colleagues reported the case of a 78 year-old man with Heyde’s syndrome, where porcine AVR wasn’t a radical treatement for his condition. Duodenal angiodysplasia has persisted endoscopically for 14 years after valve replacement. Occult bleeding resulting in significant anemia and need for transfusion had continued [7]. Giovanni and colleagues reported another 70 year-old man with chronic renal failure having Heyde’s syndrome. He underwent biological aortic valve replacement as an extreme life-saving procedure. After a bleeding-free period of 10 months, he was readmitted with severe intestinal bleeding requiring right hemicolectomy [19].

On the other hand however, and in most reported cases of Heyde’s syndrome, cessation of bleeding has been achieved with aortic valve replacement. Gastrointestinal bleeding ceases in 95% of cases, compared to only 5% in cases controled by laparotomy with or without bowel resection [14, 15]. Several case reports and a few series have reached this conclusion. Mitchell and coworkers presented in 1986 recurrent bleeding from gastrointestinal angiodysplasia in 2 elderly women with severe aortic stenosis. They reported the cessation of bleeding after aortic valve replacement, and the gradual disappearance of angiodysplasia [20]. During the same year, the case of another old woman with aortic valve stenosis suffering from recurrent gastrointestinal bleeding was reported by Scheffer and Leatherman. Despite sigmoidectomy followed by right hemicolectomy, the bleeding could only be controlled six years later by aortic valve replacement [15]. Extending these results, a series was published in 1987 by the Mayo clinic division of thoracic and cardiovascular surgery. It examined outcomes in 91 patients with chronic intestinal bleeding and aortic stenosis. Of 37 patients who had abdominal surgery, 95% continued to bleed postoperatively. The follow up ranging from 8 to 12 years of the 16 patients who underwent AVR, only one had recurrent bleeding secondary to excessive anticoagulation. They concluded that the chance of cessation of bleeding after aortic valve replacement seems to be greater than after bowel surgery [21]. Between 1988 and 2002, 4 patients with Heyde’s syndrome were reported. They all presented with significant intestinal bleeding that completely disappeared following aortic valve replacement [5,10,21,22]. One of these patients [5] experienced recurrence of melena and left ventricular failure 12 months after aortic valve surgery; the prosthetic valve was found to be stenotic. Following a second valve replacement, the bleeding disapeared despite anticoagulation. In 2003, Vincentelli and colleagues published in the New England Journal of Medicine a prospective study of 50 consecutive patients with severe aortic stenosis. Four patients experienced gastrointestinal bleeding prior to surgery. This study demonstrated that the severity of VWF deficit was directly proportional to the severity of the aortic stenosis, the percentage of high molecular weight multimers being negatively correlated with the mean transvalvular pressure gradient. One patient presented repeated epistaxis at the onset of valve restenosis. All other Patients remained asymptomatic without bleeding episodes including those with a mechanical prosthesis and oral anticoagulant therapy. Interestingly they noted that the hemostatic abnormalities tended to recur when there was a mismatch between the patient and prosthesis size [11]. 

Moreover, late recurence of intestinal bleeding has been observed following stenosis due to structural degeneration of the bioprosthesis. Interestingly, bleeding stops again following redo valve replacement [5]. These observations become even more relevant with the advent of transcutaneous aortic valve replacement in high risk and elderly patients with serious comorbidities [2]. 

Another controversial issue is the choice of the prosthesis in patients with Heyde’s syndrome [4]. Although lifelong anticoagulation is better avoided in an elderly patient with intestinal bleeding, bleeding has been reported to cease promptly following valve replacement, regardless of the type of the prosthesis, and despite the use of oral anticoagulation [2, 5, 14].

In conclusion, Heyde’s syndrome should be suspected in patients with intestinal bleeding and calcific aortic stenosis. Bleeding will stop following aortic valve replacement. In case a tissue valve is used, aortic valve restenosis usually leads to the recurrence of intestinal bleeding and resolves again after redo valve surgery.

## References

[1] Bourdette D, Greenberg B (1979). Twelve year history of gastrointestinal bleeding in a patient with calcific aortic stenosis and hemorrhagic telangiectasia. Dig Dis Sci.

[2] Mishra PK, Kovac J, Caestecker J (2009). Intestinal angiodysplasia and aortic valve stenosis: let's not close the book on this association. Eur J Cardiothorac Surg.

[3] Natwitz L, Defrelingne J, Limet R (1993). Association of aortic stenosis and gastrointestinal bleeding (Heyde's syndrome). Actra Chir Belg.

[4] Massyn M, Khan S (2009). Heyde syndrome: a common diagnosis in older patients with severe aortic stenosis. Age Aging.

[5] Soran H, Lewis M, Whorwell P (2002). Bleeding angiodysplasia: Should we concentrate more on the aortic valve than on the bowel ?. Int J Clin Pract.

[6] Sotto A, Porneuf M, Pignodel C (1993). Heyde’s syndrome: A questionnable concept. Presse Med.

[7] Bhutani MS, Gopalswamy N, Gupta SC (1994). Heyde's syndrome: The controversy continues. Am J Gastroentorol.

[8] Boss EG, Rosenbaum JM (1971). Bleeding from the right colon associated with aortic stenosis. Am J Dig Dis.

[9] Pate GE, Chandavimol M, Naiman SC (2004). Heyde's Syndrome: A review. J Heart Valve Dis.

[10] Warkentin TE, Moor JC, Morgan DG (2002). Gastrointestinal Angiodysplasia and Aortic Stenosis. N Engl J Med.

[11] Vincentelli A, Susen S, Le Tourneau T (2003). Acquired von Willebrand Syndromein aortic stenosis. N Engl J Med.

[12] Pareti Fl, Lattuada A, Bressi C (2000). Proteolysis on von Willebrand factorand shear stress-induced platelet aggregation in patient with aortic valve stenosis. Circulation.

[13] Warkentin TE, Moor JC, Morgan DG (1992). Aortic stenosis and bleeding gastrointestinal angiodysplasia: is acquired von Willebrand's disease the link ?. Lancet.

[14] King RM, Pluth JR, Guiliani ER (1987). The association of unexplained gastrointestinal bleeding in calcific aortic stenosis. Ann Thoracic Surg.

[15] Scheffer SM, Leatherman LL (1986). Resolution of Heyde's syndrome of aortic stenosis and gastrointestinal bleeding after aortic valve replacement. Ann Thoracic Surg.

[16] Panzer S, Badr Eslam R, Schneller A (2010). Loss of high-molecular-weight von Willebrand factor multimers mainly affects platelet aggregation in patients with aortic stenosis. Thromb Haemost.

[17] Sadler JE (2003). Aortic stenosis, von Willebrand factor, and bleeding. N Engl J Med.

[18] Veyradier A, Balian A, Wolf M (2001). Abnormal von Willebrand factor in bleeding angiodysplasias of the digestive tract. Gastroenterology.

[19] Giovannini I, Chiarla C, Murazio M, Clemente G, Giuliante F, Nuzzo G (2006). An extreme case of Heyde syndrome. Dig Surg.

[20] Cappell MS, Lebwohl O (1986). Cessation of recurrent bleeding from gastrointestinal angiodysplasias after aortic valve replacement. Ann Intern Med.

[21] King RM, Pluth JR, Giuliani ER (1987). The association of unexplained gastrointestinal bleeding with calcificaortic stenosis. Ann Thorac Surg.

[22] Casson AG, McKenzie NN (1988). Heyde’s syndrome. Chest.

